# Disparate Regulatory Mechanisms Control Fat3 and P75^NTR^ Protein Transport through a Conserved Kif5-Interaction Domain

**DOI:** 10.1371/journal.pone.0165519

**Published:** 2016-10-27

**Authors:** Haixia Cheng, Jessica Burroughs-Garcia, Jacqueline E. Birkness, Jonathan C. Trinidad, Michael R. Deans

**Affiliations:** 1 Department of Surgery, Division of Otolaryngology-Head and Neck Surgery, University of Utah School of Medicine, Salt Lake City, Utah, United States of America; 2 Zanvyl Krieger School of Arts and Sciences, Johns Hopkins University, Baltimore, Maryland, United States of America; 3 Department of Chemistry, Indiana University, Bloomington, Indiana, United States of America; 4 Department of Neurobiology & Anatomy, University of Utah School of Medicine, Salt Lake City, Utah, United States of America; International Centre for Genetic Engineering and Biotechnology, ITALY

## Abstract

Directed transport delivers proteins to specific cellular locations and is one mechanism by which cells establish and maintain polarized cellular architectures. The atypical cadherin Fat3 directs the polarized extension of dendrites in retinal amacrine cells by influencing the distribution of cytoskeletal regulators during retinal development, however the mechanisms regulating the distribution of Fat3 remain unclear. We report a novel Kinesin/Kif5 Interaction domain (Kif5-ID) in Fat3 that facilitates Kif5B binding, and determines the distribution of Fat3 cytosolic domain constructs in neurons and MDCK cells. The Kif5-ID sequence is conserved in the neurotrophin receptor P75^NTR^, which also binds Kif5B, and Kif5-ID mutations similarly result in P75^NTR^ mislocalization. Despite these similarities, Kif5B-mediated protein transport is differentially regulated by these two cargos. For Fat3, the Kif5-ID is regulated by alternative splicing, and the timecourse of splicing suggests that the distribution of Fat3 may switch between early and later stages of retinal development. In contrast, P75^NTR^ binding to Kif5B is enhanced by tyrosine phosphorylation and thus has the potential to be dynamically regulated on a more rapid time scale.

## Introduction

Polarized protein transport is one mechanism by which cells spatially restrict protein function to establish cellular polarity, thereby regulating tissue patterning, morphogenesis and function. In neurons, polarized transport separates pre- and post-synaptic proteins between axons and dendrites thereby enabling the directional flow of action potentials across neuronal circuits. In epithelial cells, polarized transport of transmembrane proteins to the apical or basolateral cell surfaces contributes to the function of epithelial barriers around and within organs, and facilitates the vectorial transport of solutes across the epithelial sheet. The conservation of some sorting mechanisms between neurons and epithelial cells led to historical comparisons of protein transport between these cell types [1–3].

The cellular requirements for polarized protein transport are dynamic and context dependent, and can change during the course of development or in response to extracellular cues. As a result, mechanisms regulating polarized protein transport show a correspondingly high level of plasticity. For example, during the polarized maturation of Madin-Darby Canine Kidney (MDCK) cells the apical delivery of P75 neurotrophin receptor (P75^NTR^) is initially dependent upon the Kinesin3 family motor proteins Kif1A and Kif1Bβ [4]. However as MDCK cells become more polarized, Kinesin1 becomes the primary motor transporting P75^NTR^ to the apical cell surface due to preferential binding of P75^NTR^ to the Kinesin1 family motor protein Kif5B [5]. Polarized transport can also be modulated in response to extracellular signals. For example, the poly immunoglobulin receptor (pIgR) is actively inserted and internalized at the basolateral membrane. However in response to extracellular dimeric IgA, pIgR is rapidly trancytosed from the basolateral to the apical cell surface [6]. Finally, the destination of polarized protein transport may also be regulated by alternative splicing of the transcripts encoding cargo proteins. Thus for the metabotropic glutamate receptor 1 (mGluR1), alternative splicing of *mGlur1* mRNA determines which axonal or dendritic targeting motif appears in the translated protein product [7]. Together these diverse examples demonstrate that the mechanisms regulating polarized transport are plastic and adapt to changes in cellular environment, behavior or physiology.

During the course of development, the polarized transport and asymmetric distribution of cellular proteins often precedes the morphological appearance of cellular polarity. For example, during neuronal development, P75^NTR^ is enriched in the neurite that is destined to become the axon [8], and subsequent P75^NTR^ localization to the growth cone promotes axon growth [9]. Similarly the asymmetric distribution of planar cell polarity (PCP) proteins precedes the development of polarized cellular structures like the inner ear hair cell stereociliary bundle [10] and *Drosophila* wing hairs [11]. In *Drosophila*, the core PCP pathway acts in parallel to a second planar polarity pathway that is mediated by intercellular bridges composed of the atypical cadherins Fat and Dachsous [12, 13]. While *Drosophila* Fat has not been linked to polarized protein transport, the vertebrate orthologue Fat1 is alternatively spliced, and much like mGlur1 [7], different Fat1 splice isoforms are differentially distributed throughout the cell [14]. Moreover, in these heterologous cell systems, the spatial distribution of Fat1 precedes dynamic cytoskeletal rearrangements and changes in cell shape [15, 16].

**Fig 1:**
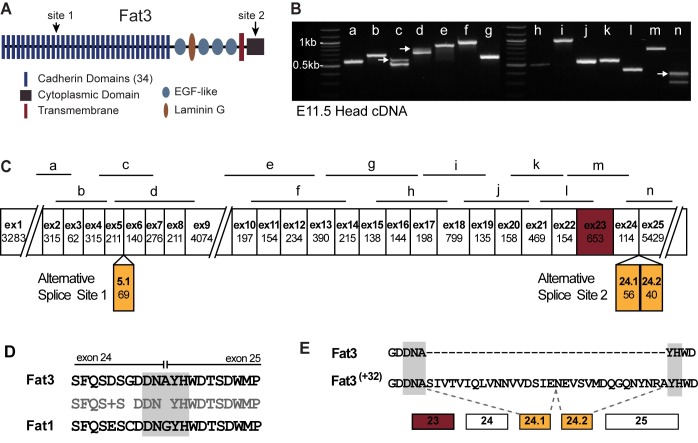
Alternative splicing of Fat3 mRNA disrupts a conserved Kif5-ID. (A) Fat3 is a single-pass transmembrane protein with 34 extracellular cadherin repeats and Laminin-G and EGF-like motifs. Numbered arrows indicate regions of the Fat3 protein modified by alternative splicing. (B) Conventional PCR products amplified from E11.5 mouse head cDNA using PCR primer pairs flanking each splicing junction predicted from Fat3 NCBI reference sequence NM_001080814.1. Arrows indicate amplification products resulting from the insertion of alternative exons. (C) Schematic of the Fat3 cDNA showing the position of each of the amplified products in (B) relative to the 25 Fat3 exons and the three alternative exons 5.1, 24.1 and 24.2. The length of cDNA encoded by each exon is presented in basepairs, and exon lengths are not drawn to scale. Exon 23 is shaded maroon and encodes the Fat3 transmembrane domain. (D) Primary amino acid sequence encoded by nucleotide sequences at the splice junction between mouse Fat3 exons 24 and 25 includes a conserved DNXYH motif (gray shading) that has been described in mouse Fat1 [14]. (E) This motif is disrupted by the insertion of 32 amino acids encoded by alternative exons 24.1 and 24.2.

The Fat cadherins are extremely large, single pass transmembrane proteins containing 34 extracellular cadherin repeats and Laminin G and EGF-like motifs (Fig 1A). The mouse genome encodes four Fat Cadherin genes [17] and Fat3 has been detected throughout retina development where it is expressed by amacrine and ganglion cells [18, 19] and directs synaptic layer formation promoting the polarized extension of amacrine cell dendrites into the Inner Plexiform Layer (IPL) [18]. Fat3 binds to the actin regulators Ena/VASP and thus may direct amacrine cell development by recruiting these proteins to the leading edge of migrating cells where they could promote dendritic projections directly into the IPL [20].

We have found that the gene structure and alternative splicing events that determine Fat1 distribution in heterologous cells are conserved during Fat3 processing, thus raising the possibility that alternative splicing events similarly regulate Fat3 distribution during retina development. Consistent with this hypothesis, we identified a conserved and developmentally regulated Kif5-Interaction domain (Kif5-ID) in the cytoplasmic region of Fat3 that mediates delivery of Fat3 to the apical surface in polarized MDCK cells and to the tips of neurites in cultured neurons. This domain is regulated by RNA processing, and the insertion of alternative exons alters Fat3 protein distribution. In addition, the essential amino acids of the Kif5-ID are present in P75^NTR^ and similarly contribute to P75^NTR^ distribution in MDCK cells. Protein interaction assays identified the Kinesin motor protein Kif5B as a specific binding partner of the Kif5-ID in both Fat3 and P75^NTR^, and that Kif5B mediates the delivery of both proteins to the apical surface of MDCK cells. Despite these similarities, the mechanisms regulating Kif5B-mediated protein transport are distinct, with the association between Fat3 and Kif5B regulated by alternative splicing while P75^NTR^ binding to Kif5B is dynamically regulated by tyrosine phosphorylation.

## Results

### Fat3 mRNA transitions between alternatively spliced isoforms during development of the mouse retina

In heterologous cells, the vertebrate Fat1 gene is alternatively spliced at a region of the RNA encoding the cytoplasmic domain and this determines the subcellular distribution of different Fat1 protein isoforms [14]. Since Fat3 has a similar gene structure in this region, and both genes contain more than 25 exons, the potential for alternative splicing of the Fat3 gene was examined by RT-PCR. PCR primers used for cDNA amplification flanked predicted intron-exon boundaries, and each Fat3 exon less than 400bp in length based upon the NCBI reference sequence NM_001080814 (Fig 1B and 1C and S1A and S1B Fig). Conventional amplification of cDNA isolated from several different mouse tissues identified two sites of alternative splicing (Fig 1 and S1C Fig). Alternative splicing at these sites involved the insertion of alternative exons not found in NM_001080814, and these additional exons do not introduce frame shifts or premature stops (Fig 1E and S1D and S1E Fig).

One site of alternative splicing occurs in a portion of the Fat3 mRNA encoding sequences adjacent to the 11^th^ extracellular cadherin repeat located between exons 5 and 6. The second site is analogous to the site of alternative splicing identified for Fat1 [14], occurring between exons 24 and 25, and encoding a region of the cytoplasmic domain conserved between the two proteins (Fig 1). Based upon their position in the Fat3 reference (NM_001080814), the alternative exons inserted at these locations have been named exon 5.1, 24.1 and 24.2, respectively (Fig 1C). Exon 5.1 is 69 bps in length while 24.1 and 24.2 consisted of two shorter 56 and 40 basepair sequences (S1D Fig). Together, exons 24.1 and 24.2 encode 32 amino acids and produce protein that is analogous to the Fat1 ^(+32)^ isoform described by Braun and colleagues [14]. Unlike Fat1, there is no evidence from tissues tested of a Fat3 isoform analogous to Fat1 ^(+12)^ containing only one additional exon and resulting in a premature stop. All conventional RT-PCR evidence suggests that Fat3 exons 24.1 and 24.2 are an obligate pair, and that alternative splicing between exons 24 and 25 occurred in all tissues tested (Fig 1B and S1C Fig). No cDNA fragments were amplified that were missing any exons predicted by NM_001080814.

**Fig 2:**
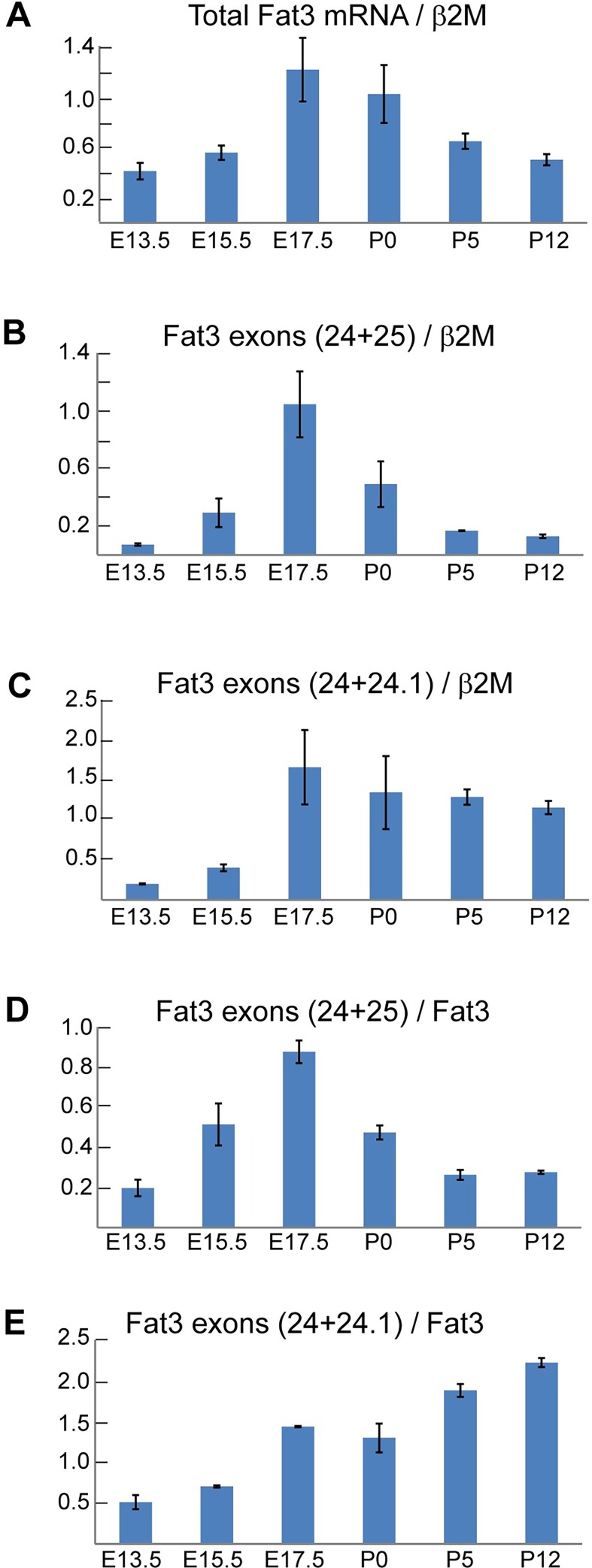
Fat3 mRNA transitions between alternative splice isoforms during the course of eye development. (A) Expression of Fat3 mRNA (all isoforms) during whole eye development assayed by Taqman® qRT-PCR relative to the β2M reference gene. (B,C) Isoform-specific Taqman® qRT-PCR reactions distinguish between Fat3 cDNA without alternative exons (24+25) and cDNA containing alternative exon 24.1 (24+24.1), and demonstrate the dynamic pattern of alternative splicing relative to the β2M reference gene. (D,E) Multiplexed Taqman® qRT-PCR reactions demonstrate the dynamic expression of different splice isoforms relative to total Fat3 mRNA. qRT-PCR results are the mean of three biological replicates and all error bars are ± SEM.

**Fig 3:**
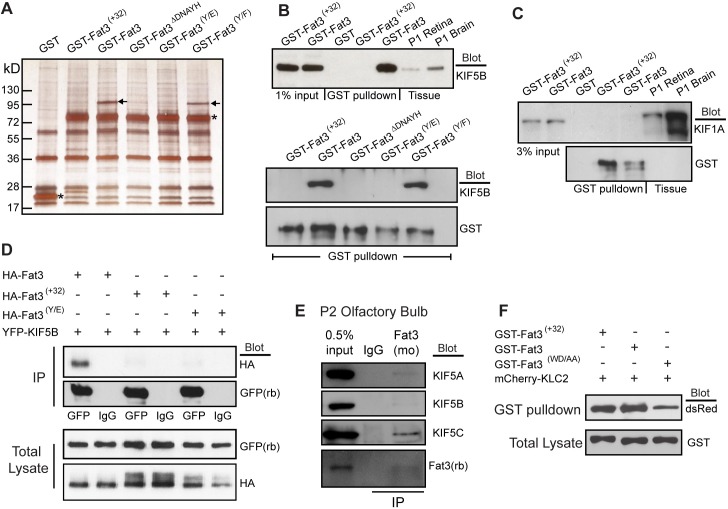
Kif5B binds to the Fat3 Kif5-ID *in vitro* and *in vivo*. (A) Silver stained gel showing proteins co-purified by GST-pulldown from HEK293 cells expressing GST-Fat3 fusion proteins corresponding to proteins encoded by alternative splice isoforms, and mutants targeting the conserved Kif5-ID. Arrows mark a unique protein that binds the Kif5-ID of GST-Fat3 and GST-Fat3 ^(Y/F)^ mutants that was identified as Kif5B by mass spectrometry analysis. Asterisks show the position of purified GST-fusion proteins. (B) Western blot analysis of GST-pulldowns from HEK293 cells using a Kif5B antibody confirms the mass spec identification and further demonstrates the specificity of Kif5B binding to different Fat3 splice isoforms and mutants. (C) Western blot using an antibody against the Kinesin3 motor protein Kif1A demonstrates Kif1A expression in HEK293 cells but no binding to Fat3. As previously reported, Kif1A occasionally runs as a dimer when detected in whole tissue lysate such as brain [41]. (D) Immunoprecipitation of YFP-Kif5B fusion protein using goat anti-GFP antibodies from HEK293 cells co-transfected with HA-tagged, membrane spanning Fat3 constructs. Immunoprecipitation using non-specific antisera (IgG) served as a negative control. (E) Immunoprecipitation of full length Fat3 from P2 mouse olfactory bulb using a mouse polyclonal antibody co-precipitated all endogenous Kif5 isoforms (Kif5A, Kif5B, Kif5C). Western blot analysis using a second, rabbit anti-Fat3 antibody demonstrated expression in this tissue and immunoprecipitation efficiency. (F) GST-pulldown of GST-Fat3 fusion proteins from HEK293 cells co-transfected with mCherry-KLC2. Western blot analysis using dsRed antibodies to detect mCherry demonstrate co-purification of mCherry-KLC2 with Fat3 regardless of splice isoform or mutation of a putative bipartite WD Kinesin binding motif.

The temporal dynamics of Fat3 alternative splicing were examined in the developing eye because Fat3 is required for development of the vertebrate retina, and Fat3 knockouts show retinal lamination and synaptic stratification deficits [18]. Isoform-specific Taqman® assays demonstrate that the overall level of Fat3 mRNA, in addition to the prevalence of alternatively spliced isoforms, is dynamic relative to a stably expressed reference gene (beta-2-Microglobulin, β2M) during embryonic and perinatal eye development (Fig 2A–2C and S1 Fig 2,3). The protein domain encoded by mRNA at the junction of Fat3 exons 24 and 25 is highly conserved between Fat1 and Fat3 and has been characterized in Fat1 as an important protein binding domain [14]. In Fat3 this sequence is disrupted by the insertion of the 32 amino acids encoded by exons 24.1 and 24.2 (Fig 1E). Fat3 mRNA isoforms encoding this domain, detected by Taqman® probes spanning the junction between exons 24 and 25 (24+25), peak at embryonic day 17 (E17) and then rapidly decline after birth relative to β2M (Fig 2B). In contrast, changes in total Fat3 mRNA are more gradual, increasing steadily throughout development and only gradually falling after birth and before eye opening (Fig 2A). This difference is due to the appearance of alternatively spliced mRNA species containing exon 24.1 at later stages (Fig 2C).

The developmental progression of Fat3 splice variants is further evident when isoform-specific Taqman® assays are evaluated relative to total Fat3 mRNA (Fig 2D and 2E). Thus Fat3 mRNA lacking alternative exons is transient and only contributes to retinal development at neonatal stages. At postnatal stages, mRNA containing exon 24.1 is predominant (Fig 2E). In addition, the low abundance of assayed splice isoforms at E13.5 raise the potential that there are other, yet to be identified, Fat3 mRNA isoforms at early developmental stages (Fig 2D). In contrast, the prevalence of exon 5.1 steadily increases relative to β2M after birth and there appears to be a binary transition between splicing outcomes in this region of the Fat3 gene (S3 Fig). Therefore, since the temporal profile of alternative splicing between exons 5 and 6, and exons 24 and 25 are different, alternative splicing at these two locations must be regulated independently.

### Alternative splicing of Fat3 mRNA regulates Fat3 protein binding to Kif5B

The developmental sequence of Fat3 alternative splicing argues that this is functionally significant and that different Fat3 protein isoforms could contribute to different aspects of retinal development. Since the cytoplasmic domain of transmembrane proteins are most likely to regulate intracellular functions the complement of proteins binding to the different cytoplasmic domains encoded by Fat3 splice variants was evaluated by GST pull-down. Fusion proteins containing glutathione S-transferase (GST) linked to the intact cytoplasmic domain of Fat3 or Fat3 ^(+32)^ were built in the eukaryotic expression vector pCMV-GST [21] and overexpressed in HEK293 cells (S4A and S4B Fig). Silver-staining of proteins co-purified with GST-Fat3 or GST-Fat3 ^(+32)^ from HEK293 lysates by GST pull-down reveals an isoform-specific binding partner of approximately 125kDa that was only co-purified from cells transfected with GST-Fat3 and not GST-Fat3 ^(+32)^ or GST alone (Fig 3A). Since this band only appeared in GST-Fat3 pulldowns, it likely binds to the region that is disrupted by the 32 amino acid insertion encoded by exons 24.1 and 24.2.

The mRNA from the homologous cadherin Fat1 is also alternatively spliced in this region, and the insertion of alternative exons disrupts a DNXYH amino acid motif (where X may be any amino acid) that is conserved between Fat1 and Fat3 (Fig 1D), and alters the sub-cellular distribution of Fat1 protein. Moreover the tyrosine (Y) residue within this motif is required for this function [14]. For Fat3, this corresponds to Y-4346 based upon translation of the NM_010080814 reference sequence. Deletion of the corresponding DNAYH sequence in Fat3 (GST-Fat3 ^ΔDNAYH^) or substitution of Fat3 Y-4346 with glutamic acid (GST-Fat3 ^(Y/E)^) prevented the co-purification of the 125kDa protein (Fig 3A and S4A and S4B Fig). In contrast, replacing Y-4346 with phenylalanine (GST-Fat3 ^(Y/F)^), an amino acid which is structurally similar to tyrosine but cannot be phosphorylated, had no effect on protein binding (Fig 3A).

The identity of the co-purified protein contained in this 125kDa band was determined by excising it from the silver stained gel and subjecting it to mass spectrometry analysis. Tandem mass spectrometry identified it as Kif5B, a kinesin heavy chain motor of the Kinesin1 protein family. Kinesin1 ATP-dependent motors are comprised of dimers of either Kif5A, Kif5B or Kif5C that carry proteins and vesicle cargos towards the plus-end of polymerized microtubule bundles [22]. Kif5B is broadly expressed while Kif5A and Kif5C are predominantly neuronal [23]. Mass spectrometry identification was validated by Western blot analysis of GST-pull downs using an antibody against Kif5B (Fig 3B). This antibody recognized a single band of the correct molecular mass in crude protein lysates from postnatal day 1 retina and brain in addition to the pull-down from HEK293 cell lysates. Furthermore, Kif5B Western blots demonstrated Kif5B co-purification with GST-Fat3 but not GST-Fat3 ^(+32)^, GST-Fat3 ^ΔDNAYH^, or GST-Fat3 ^(Y/E)^. Similar to the candidate band visualized by silver staining, the Kif5B interaction with GST-Fat3 was independent of tyrosine phosphorylation because Kif5B still bound to GST-Fat3 ^(Y/F)^ (Fig 3B). Finally, this does not appear to be a generic Kinesin interaction domain because GST-Fat3 does not interact with the Kinesin3 family member Kif1A which is found in HEK293 cells (Fig 3C). Therefore, since the conserved DNXYH sequence shared between Fat1 and Fat3 facilitates Fat3 binding to Kif5B but not to Kif1A this amino acid motif is likely part of a conserved Kif5 Interaction Domain (Kif5-ID).

The role of the Kif5-ID in facilitating the molecular interaction between Kif5B and Fat3 was further evaluated through a series of co-immunoprecipitation assays. For these studies, membrane spanning, HA-tagged Fat3 constructs containing the endogenous Fat3 signal sequence and an extracellular hemagluttinin (HA) epitope followed by the Fat3 transmembrane sequence and cytoplasmic domain were expressed in HEK293 cells (S4C and S4D Fig). Immunoprecipitation of YFP-Kif5B tagged constructs using GFP antibodies pulled-down YFP-Kif5B together with co-transfected HA-Fat3 but not HA-Fat3 ^ΔDNAYH^ or HA-Fat3 ^(Y/E)^ mutants in which the Kif5-ID is mutated (Fig 3D). Consistent with these *in vitro* interactions, immunoprecipitation of native Fat3 from postnatal mouse olfactory bulb, a tissue that expresses high levels of Fat3 [19], co-precipitated Kif5B and further demonstrated that the protein interaction also occurs *in vivo* (Fig 3E). The potential for the Kif5-ID to bind the neuronal Kinesin1 family members Kif5A and Kif5C was also demonstrated by Fat3 co-immunoprecipitation experiments from mouse olfactory bulb, and the relative levels of co-purified protein suggests that Kif5C is the primary Kinesin associated with Fat3 in this tissue (Fig 3E).

The means by which Kinesin1 binds to different protein cargoes is dependent upon the cargo with some proteins binding directly to the Kif5 motor and others associating indirectly through Kinesin Light Chains (KLC) that can form heterotetramers with the Kif5B heavy chains while binding to cargo molecules directly [22]. In some instances protein binding to the KLC intermediate is mediated by bipartite tryptophan-based motifs (WD/E) in the cargo protein [24]. Since WD sequences are present in Fat3 at amino acids 4301 and also 4348 which is adjacent to the Kif5-ID (Fig 1D) the potential for KLC to function as an intermediate between Fat3 and Kif5b was tested by co-transfecting HEK293 cells with mCherry-KLC2 and GST-Fat3 or GST-Fat3 ^(+32)^. KLC2 was used as a proxy for the other KLCs because, like Kif5B, it is broadly expressed in mouse tissues [25] and therefore is more likely to function with Kif5B in HEK293 cells. Under these conditions mCherry-KLC2 co-purified with both of the GST-Fat3 and GST-Fat3 ^(+32)^ constructs (Fig 3F). Since mCherry-KLC2 can bind to both Fat3 splice isoforms, KLC2 must not be the sole intermediate between Fat3 and Kif5b because the presence of mCherry-KLC2 in a complex with GST-Fat3 ^(+32)^ is not sufficient to recruit Kif5b (Fig 3B and 3D). While these results do not rule out the possibility that KLC2 contributes to assembling the final protein cargo complex, they do further demonstrate the necessity of an intact Kif5-ID for complex assembly. Curiously, mCherry-KLC2 also bound to a Fat3 mutant construct (GST-Fat3 ^(WD/AA)^) in which the bipartite trypothan motifs are replaced with alanines, indicating that either KLC2 associates with these GST-Fat3 constructs through a different site or the mCherry-KLC2 interaction with GST-Fat3 is due to non-specific hydrophobic interactions that are favored *in vitro* (Fig 3F). Whether this interaction can occur *in vivo* has not been determined. In summary, mass spectrometry analysis, GST pull-down, and co-immunoprecipitation assays demonstrate that Kif5B is physically associated, either directly or indirectly via an unidentified protein intermediate, to splice isoforms of the atypical cadherin Fat3 containing an intact Kif5-ID.

### Kif5B regulates the sub-cellular distribution of Fat3 proteins in polarized MDCK cells

Kinesins are molecular motors that deliver proteins, vesicles and mRNA along the polarized microtubule cytoskeleton. Different classes of kinesin motors have been identified that traffic to distinct regions of the cell or carry specific cargos. In neurons, Kif5B-containing Kinesin1 delivers proteins toward the plus-end of microtubules and therefore to axons and the distal tips of dendrites [22]. Kif5B function is not restricted to neurons, and has also been shown to function in polarized MDCK cells where it contributes to the delivery of the P75^NTR^ to the apical cell surface [5].

**Fig 4:**
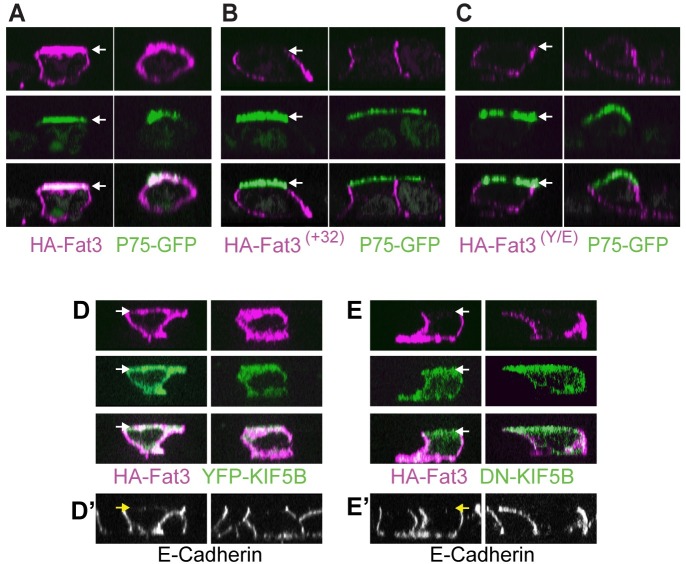
Kif5B mediates apical delivery of Fat3 in polarized MDCK cells. (A) Two examples of individual polarized MDCK cells transfected with the apical surface marker P75-GFP (green) and membrane spanning HA-Fat3 (magenta) containing an intact Kif5-ID. (B) Individual MDCK cells transfected with HA-Fat3 ^(+32)^ (magenta) show restricted distribution of HA immunolabeling at the basolateral surface. (C) Individual MDCK cells transfected with HA-Fat3 ^(Y/E)^ (magenta) similarly show restricted distribution of HA immunolabeling to the basolateral surface. (D) Pairs of individual, polarized MDCK cells transfected with membrane spanning HA-Fat3 (magenta) and full-length YFP-Kif5B (green) or (E) the dominant negative Kif5B construct DN-Kif5B in which the motor domain is replaced with GFP (green). Kif5B was visualized using antibodies directed against GFP. (A-E) For each panel, MDCK cell profiles are the orthogonal view reconstructed from Z-stacks spanning the entire height of the cell obtained by structured illumination microscopy, and for one cell in each pair the apical surface is marked by an arrow. (D’&E’) E-cadherin immunolabeling (grayscale) marks the basolateral cell surfaces demonstrating that these cells have maintained apical-basolateral polarization.

Based upon these observations, polarized MDCK cells were used as a system to test the hypothesis that selective binding of Kif5B to the Fat3 Kif5-ID could regulate the subcellular distribution of specific Fat3 splice isoforms. Fat3 is an extremely large transmembrane protein with 34 extracellular cadherin repeats. Since cadherin mediated cell adhesion may influence the distribution of Fat3 differently in epithelial versus neuronal cell lines, and because the length of the Fat3 cDNA (>14kb) makes molecular manipulations difficult, the smaller membrane spanning HA-tagged constructs were used for cellular transport assays (S4C and S4D Fig). Following transfection into polarized MDCK cells, the distribution of HA-tagged Fat3 was visualized relative to P75-GFP that was co-transfected as an apical cell surface marker [26]. Forty-eight hours after transfection, HA-Fat3 was present throughout the plasma membrane, including both basolateral and apical surfaces (Fig 4A). In contrast, HA-Fat3 ^(+32)^ and HA-Fat3 ^(Y/E)^ were only present in the basolateral membrane (Fig 4B and 4C). The apical marker P75-GFP was highly enriched at the apical cell surface in all conditions indicating that these changes in protein distribution are not secondary to changes in apical basolateral polarity.

Together these experiments suggest that a primary function of the 32 amino acid insertion is to disrupt the continuity of the Kif5-ID, thereby preventing Kif5-mediated delivery of Fat3 to specific cellular locations, which in the case of HA-Fat3 includes the apical surface of MDCK cells. This apical delivery of HA-Fat3 is dependent upon a Kif5 motor because it can be blocked by co-expression of a dominant-negative Kif5B construct (DN-Kif5B) in which the motor domain is truncated and replaced by GFP (Fig 4E) [27]. Since Kif5 proteins form heterodimers this dominant negative construct has the potential to block Kif5A and Kif5C mediated transport as well. In contrast, co-expression of functional YFP-Kif5B has no effect on the apical delivery of HA-Fat3 (Fig 4D). For these experiments E-cadherin was used as a marker for the basolateral cell surface (Fig 4D’ and 4E’) and it should be noted that in the presence of DN-Kif5B, HA-Fat3 is still delivered to this region of MDCK cells. Moreover, HA-Fat3 ^(+32)^ is delivered to this domain despite its inability to bind Kif5B. Thus while protein transport of HA-tagged Fat3 constructs to the apical surface requires Kinesin1, transport from the trans-Golgi to the basolateral cell surface is mediated by alternative motor proteins.

### A Conserved Kif5-ID contributes to the apical delivery of P75^NTR^

**Fig 5:**
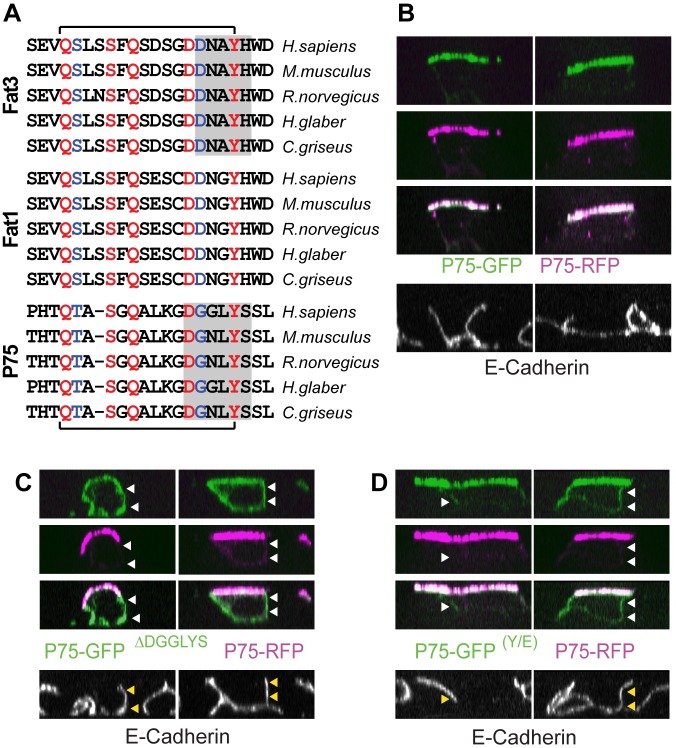
A Conserved Kif5-ID mediates the apical delivery of P75^NTR^. (A) Primary amino acid alignments of Fat3, Fat1 and P75^NTR^ protein sequences from a collection of mammalian species suggest conservation of the Fat3 Kif5-ID (bracketed sequences). Amino acids that are highly conserved between proteins and species are red, and conservative amino acid substitutions are blue. Gray shading indicates motifs in Fat3 and P75^NTR^ that have been experimentally tested. (B) Pairs of individual MDCK cells co-transfected P75-GFP (green) and P75-RFP (magenta) show colocalization at the apical cell surface. (C) Individual MDCK cells transfected with P75-GFP ^ΔDGGLYS^ in which a portion of the conserved Kif5-ID is deleted show redistribution of GFP labeling on the basolateral surface (arrowheads). (D) MDCK cells transfected with P75-GFP ^(Y/E)^ (green) show a mild redistribution of GFP labeling to the basolateral surface (arrowheads). (B-D) For each panel, MDCK cell profiles are orthogonal views reconstructed from Z-stacks obtained by structured illumination microscopy, and E-cadherin immunolabeling (grayscale) marks the basolateral cell surfaces.

P75^NTR^ is a well-characterized marker of the apical cell surface and has been used to study mechanisms of apical protein traffic [26]. Several mechanisms have been proposed for sorting apical proteins at the trans-Golgi [28] and both Kif5B and Kif5C have been demonstrated to deliver P75^NTR^ to the apical surface of polarized MDCK cells [5, 29]. Therefore Clustal V protein alignments were used to compare primary amino acid sequences between P75^NTR^ and the Fat cadherins. Alignment of Fat3, Fat1 and P75^NTR^ protein sequences from divergent mammalian species reveals a conserved amino acid sequence that includes the DNXYH motif of the Kif5-ID identified for Fat1 and Fat3 (Fig 5A). In P75^NTR^, this sequence contains Y-308 which was conserved in all sequences evaluated and corresponds to Fat3 Y-4346. Like Fat3, this sequence contributes to the apical delivery of P75^NTR^ because deleting the sequence (P75-GFP ^ΔDGGLYS^) disrupts apical delivery and results in the accumulation of GFP-tagged protein in the basolateral membrane (Fig 5B and 5C). Similarly, substituting Y-308 with glutamic acid (E) leads to the basolateral accumulation of the mutant construct (P75-GFP ^(Y/E)^) although the effect this substitution has on protein distribution is milder than the deletion (Fig 5D). Cells were co-transfected with P75-RFP to mark the apical surface and facilitate the direct comparison of mutant and wild type P75^NTR^ protein transport (Fig 5B–5D).

**Fig 6:**
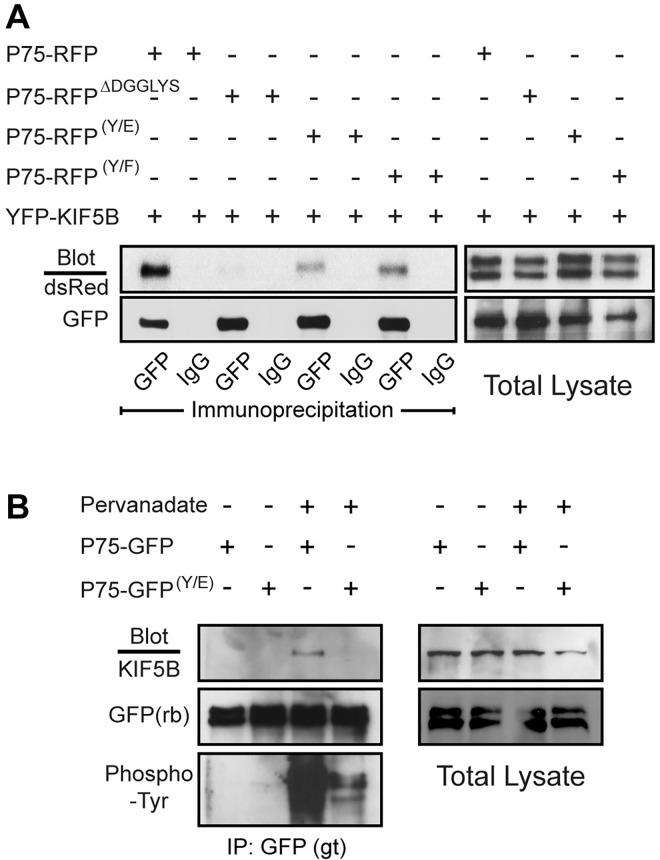
P75^NTR^ binding to Kif5B is positively regulated by Y-308 phosphorylation. (A) Immunoprecipitation of YFP-Kif5B fusion protein using GFP antibodies from HEK293 cells co-transfected with RFP-tagged P75^NTR^ constructs. Protein interactions required the conserved Kif5-ID and is lost when this domain is deleted (P75-RFP ^ΔDGGLYS^) and reduced when Y-308 is mutated (P75-RFP ^(Y/E)^) or replaced with non-phosphorylated phenylalanine. P75-RFP constructs are detected by antibodies against dsRed in Western blots, and immunoprecipitation using non-specific antisera (IgG) served as a negative control. P75-RFP doublets can be resolved with longer electrophoresis times as seen in total cell lysates. (B) HEK293 cell treatment with pervanadate promoted P75-GFP phosphorylation at Y-308 and enabled the co-precipitation of endogenous Kif5B and transfected P75-GFP. P75-GFP was precipitated using Goat (gt) anti-GFP antisera and then Western blotted using Rabbit (rb) anti-GFP to confirm that comparable amounts of P75-GFP were being evaluated for phosphorylation state and Kif5B binding in each condition. Enhanced protein binding and phosphorylation relied specifically on Y-308 and did not occur with P75-GFP ^(Y/E)^ mutants.

The P75-GFP ^ΔDGGLYS^ mutation that disrupts apical delivery of P75^NTR^ is analogous to the GST-Fat3 ^ΔDNAYH^ mutation that disrupts the Kif5-ID and prevents Fat3 binding to Kif5B in GST pull-down experiments. Co-immunoprecipitation assays demonstrate that the corresponding Kif5-ID sequence in P75^NTR^ also facilitates binding to Kif5B. Specifically, P75-RFP co-immunoprecipitates with YFP-Kif5B in HEK293 cells expressing both constructs (Fig 6A), and similarly P75^NTR^ and Kif5B can be co-immunoprecipitated from MDCK cells [5]. *In vitro*, these interactions are dependent upon the presence of conserved amino acids in the Kif5-ID of P75^NTR^ because the interaction failed to occur for P75-RFP ^ΔDGGLYS^ mutants, and is reduced when Y-308 in the Kif5-ID is replaced with glutamic acid (P75-RFP^(Y/E)^; Fig 6A). Unlike Fat3, the Kif5-ID in P75^NTR^ does not span an exon splicing junction and therefore it is not possible for P75^NTR^ protein distribution to be regulated through an alternative splicing mechanism analogous to the regulation of Fat3. Nonetheless, Kif5B mediated transport of P75^NTR^ to the apical surface of MDCK cells is dynamically regulated during epithelial cell maturation. Initially, P75^NTR^ delivery is mediated by the Kinesin3 family motor proteins Kif1A or Kif1Bβ [4]. However, after apical-basal polarization, the transport of P75^NTR^ is dependent upon Kinesin1 motors and specifically Kif5B [5]. One possibility is that, as an alternative to RNA processing, P75^NTR^ binding to Kif5B is regulated via post-translational modifications. Consistent with this hypothesis, phosphorylation of Y-308 has been reported *in vivo*. In this case, phosphorylation promoted binding to another protein, the E3 ubiquitin ligase c-Cbl [30]. While there is no evidence that c-Cbl is involved in apical transport, this report shows that Y-308 has the potential to be phosphorylated, and demonstrates that phosphorylation promotes protein binding.

The potential for Y-308 phosphorylation to regulate Kif5-ID function was evaluated in HEK293 cells transfected with P75-GFP or P75-GFP^(Y/E)^ and treated with pervanadate which prevents dephosphorylation. In the absence of pervanadate treatment, immunoprecipitation of P75-GFP using antibodies against GFP did not co-precipitate endogenous Kif5B, while co-precipitation of Kif5B was increased following pervanadate treatment (Fig 6B). Kif5B co-precipitation was correlated with dramatic phosphorylation of P75-GFP suggesting that tyrosine phosphorylation positively regulates P75^NTR^ binding to Kif5B. This correlation was seen across three separate experiments. Moreover, the regulatory phosphorylation event occurs specifically at Y-308 because P75-GFP ^(Y/E)^ is not phosphorylated to a similar extent following pervanadate treatment (Fig 6B). Thus while Y-308 is not required for P75^NTR^ to bind Kif5B, the affinity between these two proteins is enhanced by phosphorylation. Consistent with this hypothesis, YFP-Kif5B co-immunoprecipitation with a P75-RFP ^(Y/F)^ mutant that cannot be phosphorylated is reduced compared to P75-RFP (Fig 6A). Therefore, in contrast to Fat3, P75 binding to Kif5B must be regulated post-translationally, and in heterologous cell systems this regulation likely occurs at the level of Y-308 phosphorylation.

### Alternative splicing of Fat3 regulates protein delivery to distal neuronal processes

**Fig 7:**
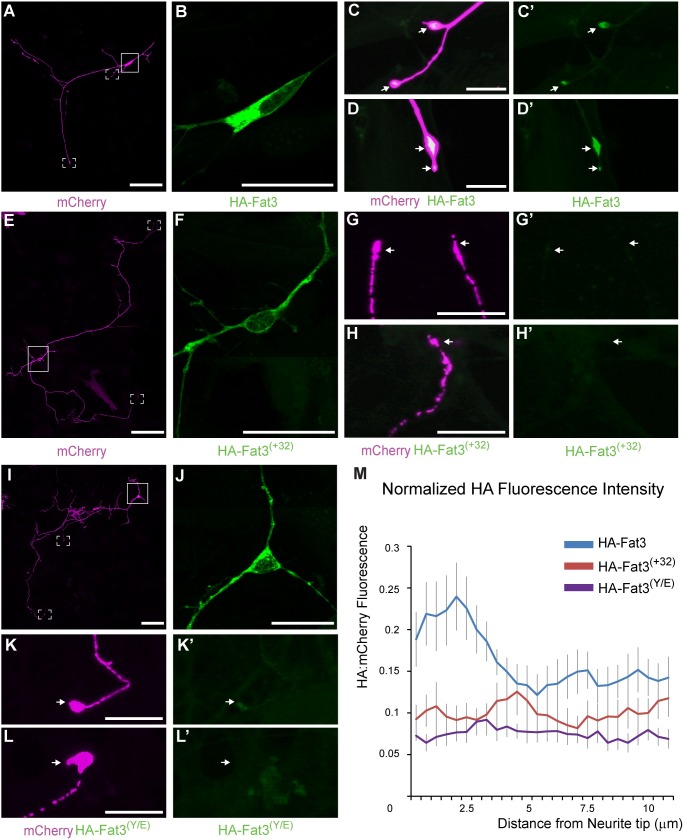
Fat3 Kif5-ID mediates protein delivery to distal neuronal process. (A,E,I) Individual cultured hippocampal neurons expressing mCherry (magenta) and HA-tagged Fat3 constructs. (B,F,J) HA immunofluorescence (green) in the cell body and proximal dendrites of transfected cells where the panels ‘B’,’F’ and ‘J’ correspond to the boxed areas from ‘A’,’E’ and ‘I’ respectively. (C,D) High magnification images of the distal tips of neuronal processes from the two framed regions in ‘A’ demonstrating the enrichment of HA-Fat3 in these locations. (G,H,K,L) High magnification images of the distal tips of neuronal processes from framed regions in ‘E’ and ‘I’. Hippocampal neurons expressing HA-Fat3 ^(+32)^ (E-H) or HA-Fat3 ^(Y/E)^ (I-L) variants in which the Kif5-ID is mutated fail to deliver HA-tagged proteins to the distal tips of neuronal processes. Arrows show the distal tip of a neuronal process. (M) Immunofluorescence intensity of HA relative to mCherry along the most distal 10um of the neuronal process, averaged from 11–16 different processes for each condition. Error bars represent standard error of the mean. Scale bars: 100μm for ‘A’,’E’ and ‘I’, and 10μm for remainder.

Protein localization studies demonstrate that Fat3 is expressed by neurons in the developing nervous system [19] and Fat3 is required for the neuronal morphogenesis during retinal development [18]. Since alternative splicing of Fat3 determines the cellular distribution of different Fat3 isoforms in MDCK cells, it seems likely that different isoforms also have distinct protein localization patterns in neurons. This hypothesis was tested by transfecting cultured hippocampal neurons with plasmids expressing membrane spanning, HA-tagged Fat3 constructs together with the fluorescent reporter mCherry to evaluate subcellular protein localization (Fig 7). In cultured neurons, HA-Fat3 was enriched at the tips of distal process and was not prominently distributed along their length (Fig 7A–7D). This distinctive spatial distribution pattern did not occur in neurons transfected with HA-Fat3 ^(+32)^ (Fig 7E–7H) or HA-Fat3 ^(Y/E)^ (Fig 7I–7L). The enhanced localization of HA-Fat3 over HA-Fat3 ^(+32)^ and HA-Fat3 ^(Y/E)^ was quantified by measuring HA immunofluorescence relative to mCherry along the most-distal 10 microns of these processes (Fig 7M). While not enriched in distal processes, HA-Fat3 ^(+32)^ and HA-Fat3 ^(Y/E)^ remained present in the cell soma and along proximal dendrites in a distribution similar to HA-Fat3 (Fig 7B, 7F and 7J). Altogether these observations from cultured neurons are consistent with the results obtained from MDCK cells, and demonstrate that alternative splicing of the Fat3 Kif5-ID regulates the sub-cellular distribution of HA-Fat3, and is likely to regulate the distribution of full length Fat3 as well.

## Discussion

We have identified a conserved Kif5-Interaction domain (Kif5-ID) that is present in the atypical cadherin Fat3 and the neurotrophin receptor P75^NTR^. This domain is essential for regulating the subcellular distribution of HA-tagged, membrane spanning Fat3 cytoplasmic domain constructs in MDCK cells and neurons, and contributes to the apical localization of P75NTR in MDCK cells. These results suggest that full length Fat3 is similarly transported to specific locations in neurons. However, the full length protein may behave differently *in vivo* if the extracellular cadherin domains also contribute to maintaining Fat3 distribution, in which case this would likely be through intercellular interactions. Through GST-pulldown and co-immunoprecipitation experiments, we demonstrate that the Kif5-ID facilitates binding to Kif5B. Furthermore, by co-expressing dominant negative Kif5B, we demonstrated that the Kinesin1 motor complex is required for Fat3 constructs to be detected on the apical surface of MDCK cells (Fig 4D and 4E) and similar experiments by Jaulin *et al*. showed the same for P75^NTR^ [5]. While these experiments do not rule out the possibility that Fat3 or P75^NTR^ associates with the other neuron-specific kinesins Kif5A or Kif5C, co-immunoprecipitation of Fat3 and Kif5B from mouse cortex confirmed the physical interaction of these two proteins in neural tissue (Fig 3D). Overall our results are consistent a function of Kif5b to ensure Fat3 and P75 protein localization at the apical cell surface, although they do not exclude the possibility that Kif5b may function to maintain this apical protein distribution rather than to deliver these proteins to this surface directly. Regardless, we propose that the Kif5-ID is a unique functional domain within diverse protein cargos that facilitates protein localization at distinct subcellular locations by binding to the Kinesin1 family motor protein Kif5B.

While the length of amino acids constituting the Kif5-ID linking specific cargos to Kif5B may be large, we have found that a single Tyrosine residue (Y-4346) is essential for Fat3 binding to Kif5B. This tyrosine is present in the sequence DNXYH that is highly conserved between Fat1 and Fat3 and was first suggested to be a phosphotyrosine binding domain (PTB) ligand because of its similarity to the PTB-ligand of Protein Kinase C3 from *C*. *elegans* [14]. However, following our identification of Kif5B as a binding partner of the Fat3 DNXYH sequence, this seems less likely because Kif5B does not contain a defined PTB domain. Furthermore the corresponding sequence in human P75^NTR^ (GGLYS) has even less sequence similarity to identified PTB-ligands than the Fat DNXYH sequences [31]. Nonetheless, cargo proteins may be linked to Kif5B through other intermediates and our data do not exclude the possibility of additional proteins linking Fat3 or P75^NTR^ to Kif5B, or the possibility that this protein contains a PTB-domain. In addition, Kinesin Light Chains may couple Kif5b to select cargoes when Kinesin1 is assembled as a heterotetramer [22]. Consistent with this possibility KLC2 can bind to GST-Fat3 *in vitro*, however binding does not require an intact Kif5-ID and KLC2 binding to GST-Fat3 ^(+32)^ is not sufficient to recruit Kif5b. One explanation is that KLC2 binding may contribute to cargo specificity, but that an intact Kif5-ID is required to recruit Kif5b, thereby facilitating Kinesin assembly and protein transport.

Based upon comparisons of the primary amino acid sequences of P75^NTR^, Fat1 and Fat3 from diverse mammalian species, we propose that the Kif5-ID is larger than the DNXYH sequence identified in Fat1 [14], and consists of the conserved amino acid sequence [**Q(S/T)**XX**S**X**Q**XXXX**D(D/G)**XX**Y**]. The carboxyl end of this sequence contains the conserved tyrosine residue that is found in the DNXYH sequence of Fat1 and Fat3, which is likely a smaller yet highly-conserved motif within the larger Kif5-ID that is required for Kif5B binding. The alternative splicing of Fat1 and Fat3 is consistent with this hypothesis because insertion of 32 amino acids into the DNXYH motif of Fat3 isolates this essential Y from the remainder of the Kif5-ID and inhibits Kif5B binding. Similarly, in a P75^NTR^ mutant used to study apical transport, the amino-terminal portion of the Kif5-ID is deleted and mutant P75^NTR^ is redistributed to the basolateral surfaces of MDCK cells. It was proposed that this disrupted apical delivery because Y-308 was repositioned in charged environment that promoted endocytosis rather than transcytotic delivery to the apical surface [26]. As an alternative we propose that the deletion disrupts the conserved Kif5-ID and the mutant protein is no longer transported to the apical surface by Kif5b. Finally, a Pattern Hit Initiated Blast (PHI-BLAST) interrogation of the human protein database (NCBI) and pair-wise BLAST analyses failed to identify additional proteins containing the Kif5-ID. However these analyses do demonstrate that the Kif5-ID is distinct from a Kinesin Binding (KB) domain identified in the Kinesin1 cargo protein RanBP2 [32, 33] and argues that Fat cadherin and P75^NTR^ interactions with Kif5B occurs through an independent protein binding site.

Despite similarities between Fat3 and P75^NTR^, one important difference between these proteins are the molecular mechanisms regulating their transport and sub-cellular distribution, specifically at the level of Kif5B binding to their respective Kif5-IDs. For Fat3, binding is strictly regulated by alternative splicing during RNA processing. As a result, Fat3 mRNA encoding an intact Kif5-ID is only present during a brief period of late embryonic and early postnatal eye development. At later postnatal ages, the insertion of two alternative exons disrupts the Fat3 Kif5-ID which should significantly alter the subcellular distribution of the resulting Fat3 ^(+32)^ isoform. In contrast, the association between P75^NTR^ and Kif5B is regulated post-translationally with enhanced protein interaction following phosphorylation of Y-308 in the Kif5-ID. Consistent with this hypothesis, P75^NTR^ Y-308 phosphorylation has been demonstrated in cell lines and rat brain [30], and YFP-Kif5B binding to P75-RFP is reduced when this tyrosine is replaced by the structurally similar amino acid phenylalanine that cannot be phosphorylated (Fig 6A). In contrast, pervanadate treatment dramatically increased P75^NTR^ Y-308 phosphorylation while also enhancing P75-GFP binding to endogenous Kif5B (Fig 6B). An important distinction between these two regulatory mechanisms is that post-translational modifications can be highly dynamic while RNA processing permanently impacts the translated protein product. The significance of this regulatory difference may be cell or tissue specific. For example, in polarized epithelial cells rapid changes in polarized protein transport may be advantageous since these cells are continually dividing. In contrast post-mitotic cells like retinal neurons are more likely to benefit from distinct transitions between protein isoforms which could have distinct developmental versus homeostatic functions. The temporal sequence of Fat3 splicing in the retina is also likely to be related to a broader transition in RNA processing that occurs during this period of retinal maturation and modulates the splicing of hundreds of genes [34].

One distinctive feature of the Fat3 mutant phenotype is the loss of neuronal polarity in developing amacrine cells. These neurons lack axons but are still uniquely polarized due to dendrites extending exclusively from the side of the cell facing the IPL and projecting directly into this synaptic layer. In the absence of Fat3 additional dendrites are formed that extend from the opposite side of the cell and project towards the outer retina resulting in an unusual ‘bipolar’ dendritic morphology [18]. This phenotype is likely to result from spatially unrestricted activities of Ena/VASP; actin regulators that bind to Fat3 and as a result are recruited to the to the leading processes of the migrating amacrine cell where they promote dendrite growth [20]. BrdU birthdating of the neonatal mouse retina reveals that the early stages of amacrine cell development match the expression profile of Fat3 mRNA encoding the Kif5-ID, with both events peaking at E17.5 (Fig 2 and [35]). Together these observations support a hypothetical model in which Fat3 transport to the leading processes of developing amacrine cells could help to corral Ena/VASP activity in this region thereby promoting asymmetric dendritic growth. While our experiments using MDCK cells and hippocampal neurons *in vitro* are consistent with this model it still requires *in vivo* validation and should be tested using mutants in which Kinesin-mediated Fat3 transport or Fat3 binding to Ena/VASP is disrupted.

Polarized protein transport is an important aspect of the development of neuronal polarity and axon specification. For example in developing neurons P75^NTR^ is enriched in the neurite that is destined to become the axon, and in the absence of P75^NTR^ neurons fail to extend axons *in vitro* and *in vivo* [8]. While these phenotypes raise the possibility that asymmetrically distributed P75^NTR^ directs neuronal morphology, it is yet to be established how P75^NTR^ becomes enriched in a single neurite. One possibility raised by the current study is that P75^NTR^ is delivered to neurites destined to become axons by Kinesin1 motors. Consistent with this, Kif5 proteins are enriched in axons [36] and similar to P75^NTR^, constitutively active Kif5C accumulates in the growth cone of nascent axons during specification of neuronal polarity [37]. Moreover while Kif5B is broadly expressed, it is significantly upregulated in neurons during axonal outgrowth [23]. One possibility is that the conserved Kif5-ID that we have identified in Fat3 and P75^NTR^ functions to link these proteins to the Kinesin1 motor for delivery to specific sub-cellular locations during neuronal development.

Since Kinesins are plus-end directed motors that travel along microtubule bundles, one requirement for these models is a pre-existing and polarized microtubule network. In neurons, the plus end of microtubules are oriented towards the growth cone. Within the retina, Fat3 and Kif5b are both expressed by retinal ganglion cells, thus raising the possibility that Fat3 protein containing an intact Kif5-ID is specifically delivered into the axon of developing retinal ganglion cells as they project towards central targets [18, 19]. In contrast to their organization in axons, the microtubule bundles in dendrites, and hence throughout the processes of amacrine cells, are oriented in both directions with plus end stabilization occurring at both proximal and distal sites [22]. In dendrites, the movement of kinesins towards distal ends is thought to be generated through the post-translational modifications of the microtubules, resulting in unidirectional kinesin movements along one track [38]. Similar modifications are proposed to facilitate kinesin mediated transport to the apical surface of MDCK cells [39]. Thus an important hypothesis resulting from our *in vitro* studies is that the development of polarized neuronal morphologies is influenced by the intracellular transport of P75^NTR^ or Fat3 to specific sub-cellular locations by Kif5B.

## Materials and Methods

### RT-PCR and Taqman® assays

For all RT-PCR applications RNA was extracted from tissues using Trizol (Life Technologies), quantified using a ND-1000 spectrophotometer (Nanodrop Technologies), and cDNA was generated using random hexamers and Super Script III RT (Life Technologies). PCR primers for conventional RT-PCR amplification were generated using LaserGene Primer Select software (DNAstar) using the NCBI NM_010080814 Fat3 reference sequence as a template. cDNA was amplified using Phusion polymerase (New England Biolabs) using C1000 thermocycler (BioRad). Detailed primer information is available as supporting information (S1 Fig). Quantitative RT-PCR was completed using Custom Taqman® assays specific for the four alternatively spliced variants or a generic reaction predicted to detect all Fat3 mRNA (total-Fat3) as illustrated (S2A Fig). RNA was extracted from three biological replicates at each developmental stage and the amplifications reactions from each consisted of three technical replicates. To facilitate multiplex qRT-PCR reactions the pan-Fat3 probe was labeled with VIC (a proprietary Life Technologies fluorophore) while the remaining probes were labeled with 6-carboxyfluorescein (FAM). Pre-made β2M reference assay (Cat.# 4331182) was FAM labeled and only used for simplex reactions. β2M reference gene used for Taqman® assays was selected by pre-screening 32 candidates from Taqman® Array Endogenous Control 96-well plate at different stages of eye development and then validate using individual reactions at additional timepoints (S2B Fig). All qRT-PCR amplifications were conducted using Applied BioSystems StepOne Plus thermocycler and Taqman® mastermix. Because preliminary amplifications demonstrated that the β2m and pan-Fat3 control reactions did not amplify with the same efficiency as the isoform-specific Fat3 assays the relative standard curves method was used for quantification [40]. All Taqman® and Applied BioSystems reagents were purchased through Life Technologies.

### Mass spectrometry

Individual candidate bands were excised from silver stained gels, diced into 1 mm squares and washed in 25 mM ammonium bicarbonate. Cysteine residues were reduced by incubating for 45 min at 57°C with 2.1 mM dithiothreitol. These reduced side chains were then alkylated with 4.2 mM iodoacetamide for 1 h in the dark at 21°C. A solution containing 12 ng/μL trypsin, in 25 mM ammonium bicarbonate was added to cover the gel pieces and the samples were digested for 12 hours at 37°C. The resulting peptides were extracted with two aliquots of 50% acetonitrile, 5% formic acid. The samples were dried down and injected into an Eksigent HPLC coupled to a LTQ Velos mass spectrometer (Thermo Fisher Scientific, Waltham MA) operating in “top eight” data dependent MS/MS selection. The peptides were separated using a 75 micron, 15 cm column packed in-house with C18 resin (Michrom Bioresources, Auburn CA) at a flow rate of 300 nl/min. A one hour gradient was run from Buffer A (2% acetonitrile, 0.1% formic acid) to 60% Buffer B (100% acetonitrile, 0.1% formic acid). MS/MS peaklists were searched against the Uniprot Homo Sapiens database (downloaded 03/21/2012) using Protein Prospector (v5.10.15).

### Plasmid constructs

Eukaryotic expression vectors containing GST-Fat3 fusions (pCMV-GST-Fat3) were generated by RT-PCR amplification of the Fat3 cytoplasmic domain from embryonic mouse tissues using Phusion polymerase (NEB) followed by TOPO-cloning into the pCRII-blunt vector (Life Technologies) and sequencing to exclude PCR-induced mutations and distinguish splice isoforms. The cytoplasmic domain of Fat3 was then sub-cloned in-frame into the pCMV-GST vector (Robert Y.L. Tsai and Randall R. Reed, 1997) using Sal1 and HindIII sites. GST-Fat3 variants were generated by synthesizing double stranded DNA containing the Fat3 Kif5-ID deletion or point mutations (Integrated DNA Technology, Coralville, Iowa) and inserting them into pCMV-GST-Fat3 between BspE1 and EcoRV sites. pCMV-HA-Fat3 constructs were generated by synthesizing double stranded DNA containing the Fat3 signal sequence, an HA epitope tag and Fat3 transmembrane domain, and using this sequence to replace GST in pCMV-GST-Fat3 constructs using EcoR1 and Ale1 sites. For neuronal expression, pCAG-MCS and pCAG-mCherry plasmids containing the chicken actin promoter were provided by Megan Williams (University of Utah). pCAG-HA-Fat3 constructs were generated by PCR amplification from pCMV-HA-Fat3 plasmids to introduce unique restriction sites and then sub-cloned by standard techniques into pCAG-MCS. The P75-GFP plasmid was provided by Geri Kreitzer (Cornell University) and P75-RFP was obtained from Addgene (plasmid #24092). Both plasmids contained the human P75^NTR^ sequence. P75-RFP variants were generated by synthesizing double strand DNA fragments of P75-RFP containing the Kif5-ID deletion or point mutations and inserting them into P75-RFP between pfIM1 and BamH1 sites. YFP-KIF5B and Dominant negative GFP-KIF5B constructs were provided by Don Arnold (University of Southern California). mCherry-KLC-N-18 construct was obtained from Addgene (plasmid # 62747). All restriction endonucleases were purchased from NEB.

### Cell culture and transfection

HEK293 cells (ATCC, #CRL-1573) were grown in MEM supplemented with 10% fetal calf serum (FCS) and 200units/ml penicillin and streptomycin. MDCK cells (Sigma #84121903) were grown in EMEM supplemented with 10% FCS, 1% nonessential amino acids, 2mM Glutamine and 200units/ml penicillin and streptomycin, and polarized MDCK monolayers were generated by plating 2.5x 10^5^ cells/well in 24-well Transwell polycarbonate filters (Costar, Cambridge, MA). For pervanadate treatment, HEK293 cells were transfected, serum-deprived in DMEM medium with 0.1% FCS for 24 hours, and then exposed to 30μM pervanadate added to the cell culture medium for 30 min. Following pervanadate treatment cell lysates were harvested and subjected to immunoprecipitation and immunoblotting as described. P0 hippocampal neurons were provided by Megan Williams (University of Utah) and grown in Neurobasal media (Invitrogen) containing 1X B27 supplement (Invitrogen), 200mM Glutamine (Invitrogen) and 200units/ml penicillin and streptomycin on a glial feeder layer. All cell types were transfected using Lipofectamine 2000 (Invitrogen) and were harvested or analyzed 48–72 hours post-transfection. For MDCK cells grown on 24-well transwell filters, 0.6ug of plasmid DNA (1.2ug total for co-transfections) was combined with 2ul of Lipofectamine 2000 in 200ul total volume of Opti-MEM (Thermo, #31985) and added directly to the growth media. For HEK293 cells grown in 6-well plates, 2.5ug of plasmid DNA was combined with 2.5ul of Lipofectamine 2000 in 1ml total volume of Opti-MEM and added directly to the growth media. For dissociated hippocampal neurons, cells were transfected after 5 days growth *in vitro* in 12-well plates using 0.75ug of plasmid DNA (1.5ug total for co-transfections) combined with 1.5ul of Lipofectamine 2000 in 150ul total volume of Opti-MEM. Neuronal growth media was replaced with 1ml Opti-MEM and transfections were completed in triplicate by adding 50ul of the Lipofectamine/DNA mixture to each well. Neurons were incubated for 2.5 hours, rinsed with fresh Opti-MEM and returned to normal growth media. Since overexpression can be highly heterogeneous, for protein localization studies MDCK cells expressing large amounts of protein throughout intracellular organelles were excluded from analysis. Imaging was restricted to cells in which transfected protein products on the cell surface.

### GST pull-down and Western blot

pCMV-GST-Fat3 constructs were transiently transfected into HEK293 cells, and cells were harvested 72 hours later with a lysis buffer containing 20mM Tris-HCl pH 8.0, 137mM NaCl, 2mM EDTA, 1% NP-40 and 10% Glycerol supplemented with Protease Inhibitor Cocktail (Sigma P8340). Cleared cell lysates were incubated with 50% slurry of Glutathione Sepharose 4B (GE Healthcare Life Sciences) for 2h at 4°C, washed four times with lysis buffer, and eluted by boiling in SDS-PAGE sample buffer. Following polyacrylamide gel electrophoresis on 4–10% gradient gels proteins were visualized by silver staining (GE Healthcare Life Sciences). For Western Blot, proteins samples were resolved by SDS-PAGE, and transferred to nitrocellulose membranes (BioRad) using a transfer buffer containing 25mM Tris and 192mM glycine. For analyzing full length Fat3, transfer buffer was supplemented with 0.05%SDS and 7.5% methanol. Blots were blocked with Tris-buffered saline, 5% non-fat dry milk and 0.05% Tween-20, and incubated with primary antibody (0.5–1ug/ml) overnight, followed by horseradish peroxidase conjugated secondary antibodies (BioRad). Immunoreactive protein bands were detected using ECL 2 western blotting substrate (Pierce).

### Immunoprecipitation

Plasmid constructs were cotransfected into HEK293 cells and 48 hours later washed twice with PBS and lysed in ice-cold lysis buffer containing 50mM Tris-HCl pH 8.0, 150mM NaCl, 1mM EDTA, 1% NP-40, and 10% Glycerol supplemented with Protease Inhibitor Cocktail (Sigma). Cell lysates were cleared by centrifugation (15,000×*g*, 15 min.) and incubated with antibodies or control IgG overnight at 4°C. Antibody bound complexes were purified using Dynabeads Protein G beads (Life Technologies) at 4°C and washed three times with lysis buffer. For immunoprecipation from tissues, lysates were pre-cleared using Protein G beads prior to the addition of antibodies or control IgG. Following purification immunocomplexes were eluted by boiling in SDS sample buffer prior to Western Blot.

### Immunofluorescence

Cells plated on trans-well filters or glass coverslips were fixed with 4% paraformaldehyde in PBS, permeabilized with 0.5% Triton X-100 in PBS for 10 min, and blocked in a solution of 10% donkey serum and 1% BSA in PBS for 1 hour. Primary antibodies were diluted in blocking solution and applied for 1 hour at room temperature, washed three times with PBS, and exchanged with secondary antibodies diluted in 1% BSA/PBS. For nuclear staining cells were incubated with 1uM DAPI (Roche) for 5 min after antibody labeling. Cells images were acquired by structured illumination microscopy using a Zeiss Imager.M2 with the Apotome.2 attachment and 63x or 100X oil objectives. Orthogonal views of MDCK cells were generated from image stacks using ZEN software (Zeiss). The relative intensity of Fat3 immunofluorescence in hippocampal neurites was measured by normalizing against total fluorescence in neurons co-transfected with mCherry using the formula (relative intensity = green/(green+red)) for each pixel along a 10μm length of distal neurite. Pixel intensity and neurite length were measured using ImageJ software (NIH) and graphed in Microsoft Excel. All images were captured with the same exposure time, and the exposures were limited to prevent pixel saturation. Normalized fluorescence values were averaged for five adjacent pixels and graphed relative distance of the central pixel from neurite tip. Graphed data is presented as Average (±SEM) for HA-Fat3 (n = 11), HA-Fat3 ^(+32)^ (n = 16), and HA-Fat3 ^(Y/E)^ (n = 11).

### Antibodies

The following primary antibodies were used in this study: Rabbit anti-dsRed (Clontech #632496), Rat anti-E Cadherin (Life Technologies #13–1900), Rabbit anti-Fat3 and polyclonal Mouse anti-Fat3 (Lisa Goodrich, Harvard Medical School [18]), Goat anti-GFP (Abcam #5450, IF and WB), Goat anti-GFP (Abcam #6673, IP only), Rabbit anti-GST (Cell Signaling Technology #2625S) Mouse anti-HA (Covance #MMS-101P), Mouse anti-Kif1A (BD Biosciences #612094), Rabbit anti-Kif5B (Thermo # PA1-642), Goat anti-Kif5B (Imgenex #IMG-3049), Rabbit anti-Kif5C (Acris # SP5236P), Mouse anti-Phospho-Tyrosine (Cell Signaling Technology #9411). Fluorescent, Dylight conjugated secondary antibodies were purchased from Jackson Immunoresearch, and HRP conjugated secondary antibodies used for Western blot were from BioRad. Whole IgG negative controls for IP experiments were from Sino Biological (Goat IgG #CR2-500) and Millipore (Mouse IgG #12–371).

### Protein sequence alignment and PHI-BLAST

Fat3, Fat1 and P75^NTR^ primary amino acid sequences were aligned using the MegAlign Sequence Analysis Software V.9.1.0 (DNAStar). Alignments were made from an amino acid sequence approximately 100 amino acids long centered upon the Kif5-ID using the Clustal V method. Following initial alignment gaps were manually removed from P75^NTR^ sequences to align the essential tyrosine residue within the Kif5-ID. The following amino acid sequences were obtained from NCBI protein databases for alignment: Fat3 (NP_001008781, NP_001074283, BAB86869, XP_004838077, XP_003499910), Fat1 (NP_005236, NP_001074755, NP_114007, XP_004853119, XP_003507507), P75^NTR^ (NP_002498, AAH38365, NP_036742, EHB16469, EGW01774). PHI-BLAST searches were similarly conducted using a 100 amino acid query sequences from Fat3, Fat1 and P75^NTR^ centered upon the conserved Tyrosine residue and the patterned hit sequence Qx(2,3)SxQx(4)Dx(3)Y. PHI-BLAST was restricted to the human protein databases.

## Supporting Information

S1 Fig Conventional PCR amplification strategy. (A) Distribution of conventional PCR amplification products relative to exons and splicing junctions in the Fat3 mRNA. This schematic is reproduced in part from Fig 1C. (B) Table summarizing the RT-PCR primer sequences, and predicted sizes of PCR amplification products based upon reference sequence NM_001080814. (C) Conventional PCR products amplified from E15.5 inner ear and P3 whole eye cDNA. Arrows indicate the position of amplified products containing alternative exons. (D) Nucleotide sequence of Fat3 alternative exons 5.1, 24.1 and 24.2. (E) Amino acid sequence encoded by alternative exon 5.1.
(TIF)

S2 Fig Isoform-specific Fat3 Taqman® qRT-PCR amplification strategy. (A) Taqman primer (arrows) and probe targets at Fat3 splicing junctions. Green bars represent FAM-labeled probes and Red bar indicates VIC-labeled probe for Total Fat3 that was used in multiplex reactions. (B) 48 Taqman® qRT-PCR amplification curves of β2M generated from equivalent amounts of RNA isolated from E13.5, E15.5, E17.5, P0, P5 and P12 whole eyes demonstrates equivalent amount of β2M mRNA at each developmental stage and validates the selection of β2M as a reference gene. All error bars are ± SEM.
(TIF)

S3 Fig Dynamic Expression of Fat3 Alternative Exon 5.1 during eye development. (A,B) Isoform-specific Taqman® qRT-PCR reactions distinguish between Fat3 cDNA without alternative exons (5+6) and cDNA containing alternative exon 5.1 (5+5.1), and demonstrate the dynamic pattern of alternative splicing relative to the β2M reference gene. (C,D) Multiplexed Taqman® qRT-PCR reactions demonstrate the dynamic expression of different splice isoforms relative to total Fat3 mRNA.
(TIF)

S4 Fig Experimental constructs and demonstration of expression in heterologous cells. (A) Schematic representation of GST-Fat3 fusion proteins used for protein purification and binding assays in HEK293 cells. (B) Western blot of cell lysates containing GST and GST-Fat3 fusion proteins with anti-GST and anti-Fat3 antibodies. (C) Schematic representation of truncated, HA-tagged Fat3 constructs containing the Fat3 signal sequence, transmembrane and cytoplasmic domains. (D) Western Blot of cell lysates with anti-Fat3 and anti-HA antibodies showing expression of HA tagged Fat3 in MDCK and HEK293 cells. (E) Schematic representation of P75^NTR^ constructs tagged with GFP or RFP. (F) Western Blot with anti-GFP antibody shows the expression of P75-GFP variants in MDCK cells. Western Blot using anti-dsRED antibody shows the expression of P75-RFP variants in HEK293 cells (reproduced from Fig 6A).
(TIF)

## Abbreviations

Kif5-ID: Kif5 Interaction Domain
P75^NTR^: P75 neurotrophin receptor
