# Supplementary figures and images for: Disparate Regulatory Mechanisms Control Fat3 and P75NTR Protein Transport through a Conserved Kif5-Interaction Domain
Source: PLoS One. 2016 Oct 27;11(10):e0165519. doi: 10.1371/journal.pone.0165519 (PMC5082931; doi:10.1371/journal.pone.0165519)

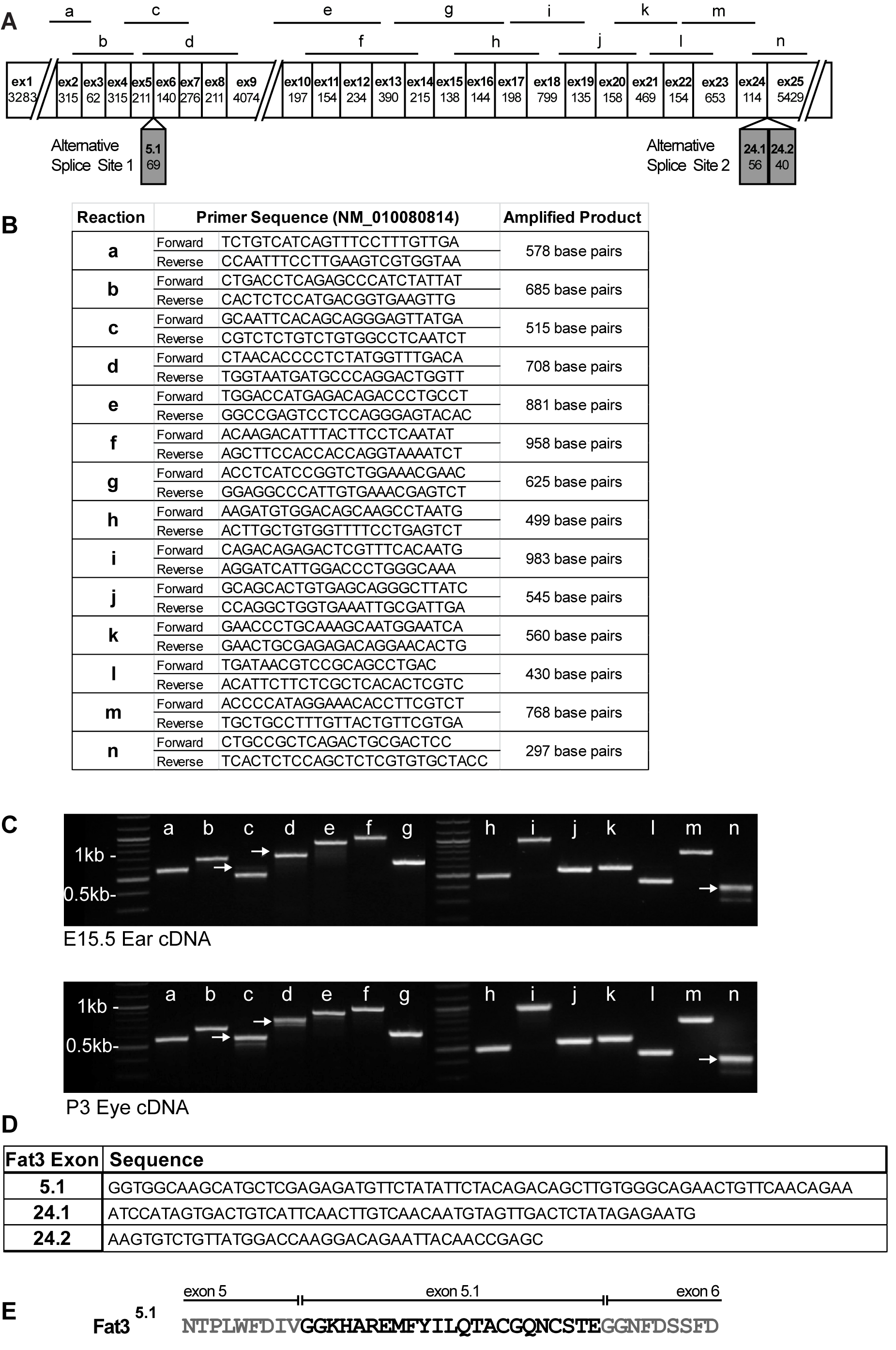

Supplement: S1 Fig — (A) Distribution of conventional PCR amplification products relative to exons and splicing junctions in the Fat3 mRNA. This schematic is reproduced in part from Fig 1C. (B) Table summarizing the RT-PCR primer sequences, and predicted sizes of PCR amplification products based upon reference sequence NM_001080814. (C) Conventional PCR products amplified from E15.5 inner ear and P3 whole eye cDNA. Arrows indicate the position of amplified products containing alternative exons. (D) Nucleotide sequence of Fat3 alternative exons 5.1, 24.1 and 24.2. (E) Amino acid sequence encoded by alternative exon 5.1. (TIF) [file pone.0165519.s001.tif]

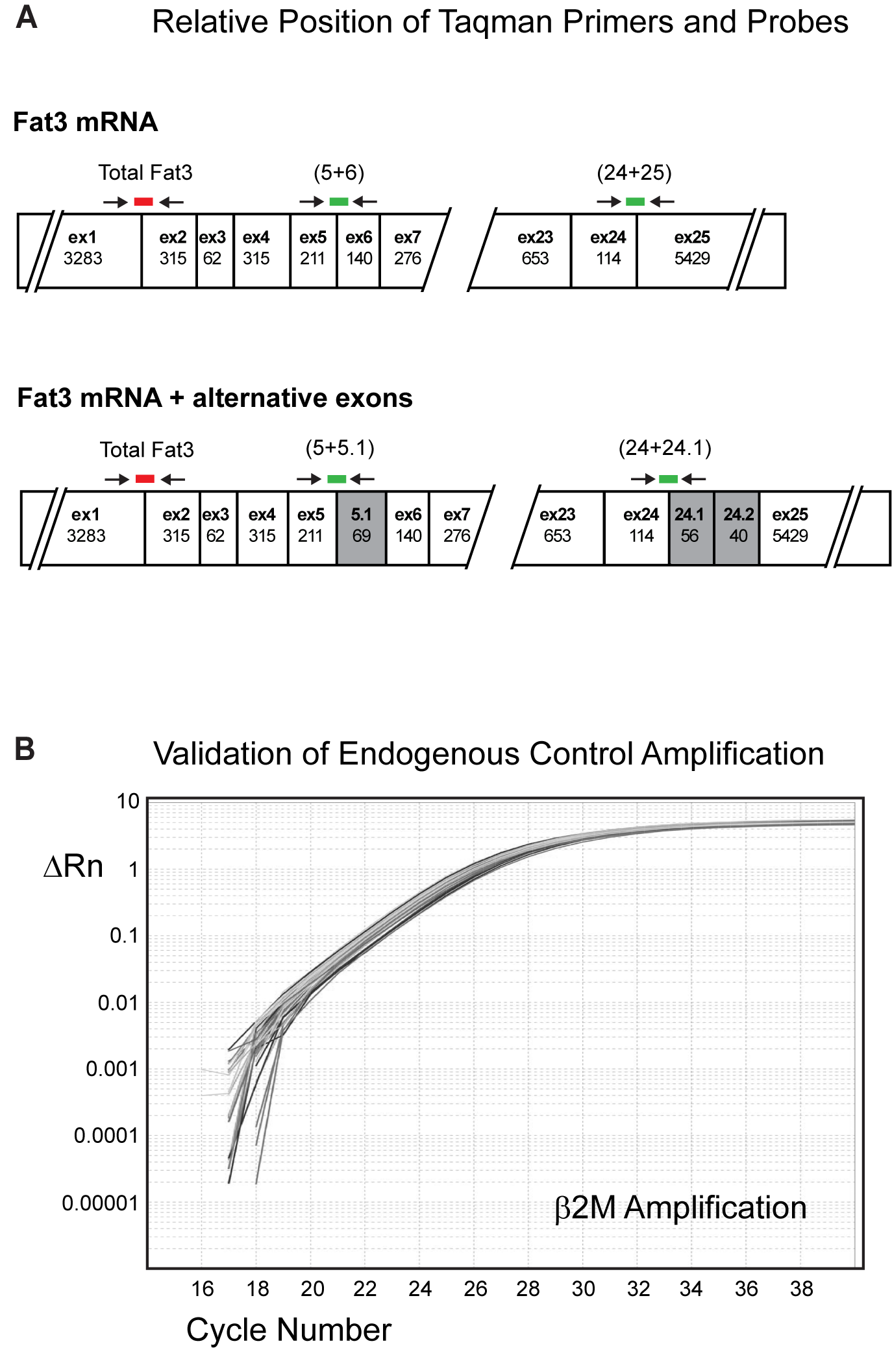

Supplement: S2 Fig — (A) Taqman primer (arrows) and probe targets at Fat3 splicing junctions. Green bars represent FAM-labeled probes and Red bar indicates VIC-labeled probe for Total Fat3 that was used in multiplex reactions. (B) 48 Taqman® qRT-PCR amplification curves of β2M generated from equivalent amounts of RNA isolated from E13.5, E15.5, E17.5, P0, P5 and P12 whole eyes demonstrates equivalent amount of β2M mRNA at each developmental stage and validates the selection of β2M as a reference gene. All error bars are ± SEM. (TIF) [file pone.0165519.s002.tif]

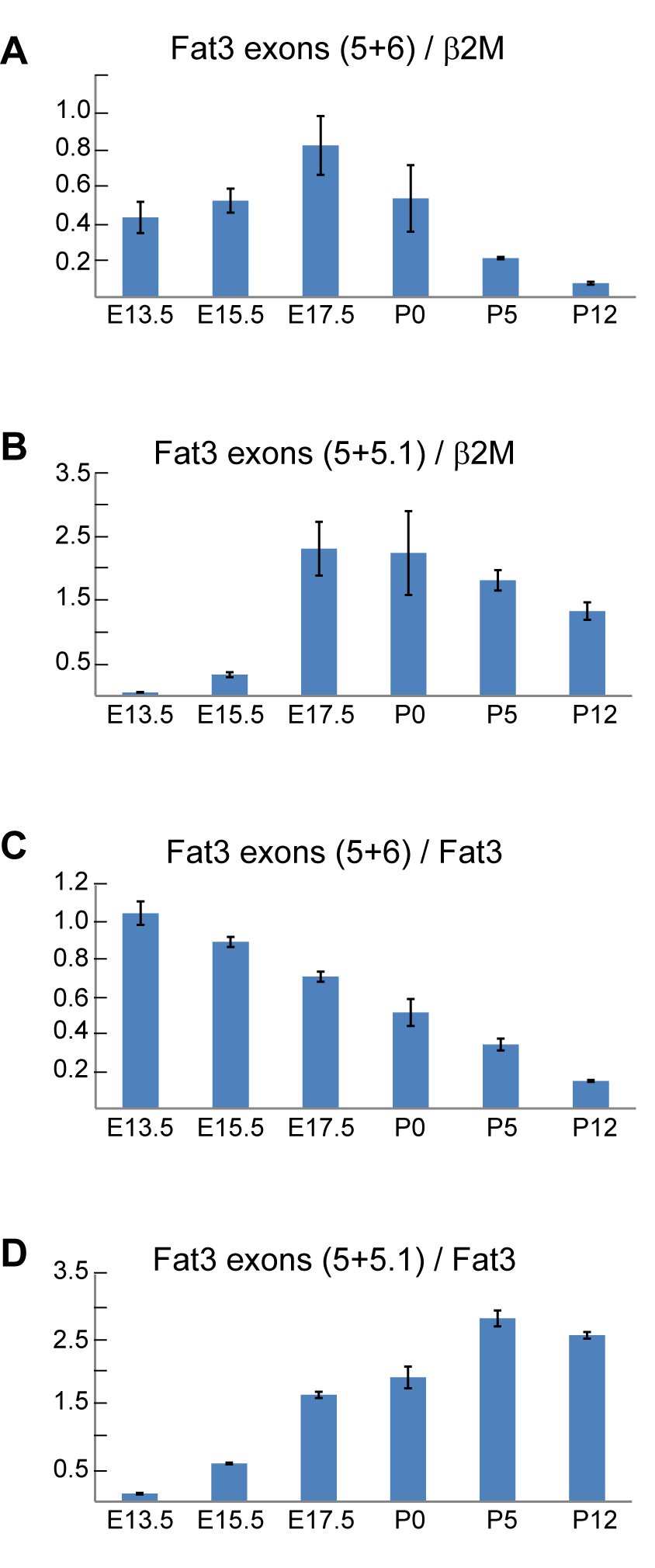

Supplement: S3 Fig — (A,B) Isoform-specific Taqman® qRT-PCR reactions distinguish between Fat3 cDNA without alternative exons (5+6) and cDNA containing alternative exon 5.1 (5+5.1), and demonstrate the dynamic pattern of alternative splicing relative to the β2M reference gene. (C,D) Multiplexed Taqman® qRT-PCR reactions demonstrate the dynamic expression of different splice isoforms relative to total Fat3 mRNA. (TIF) [file pone.0165519.s003.tif]

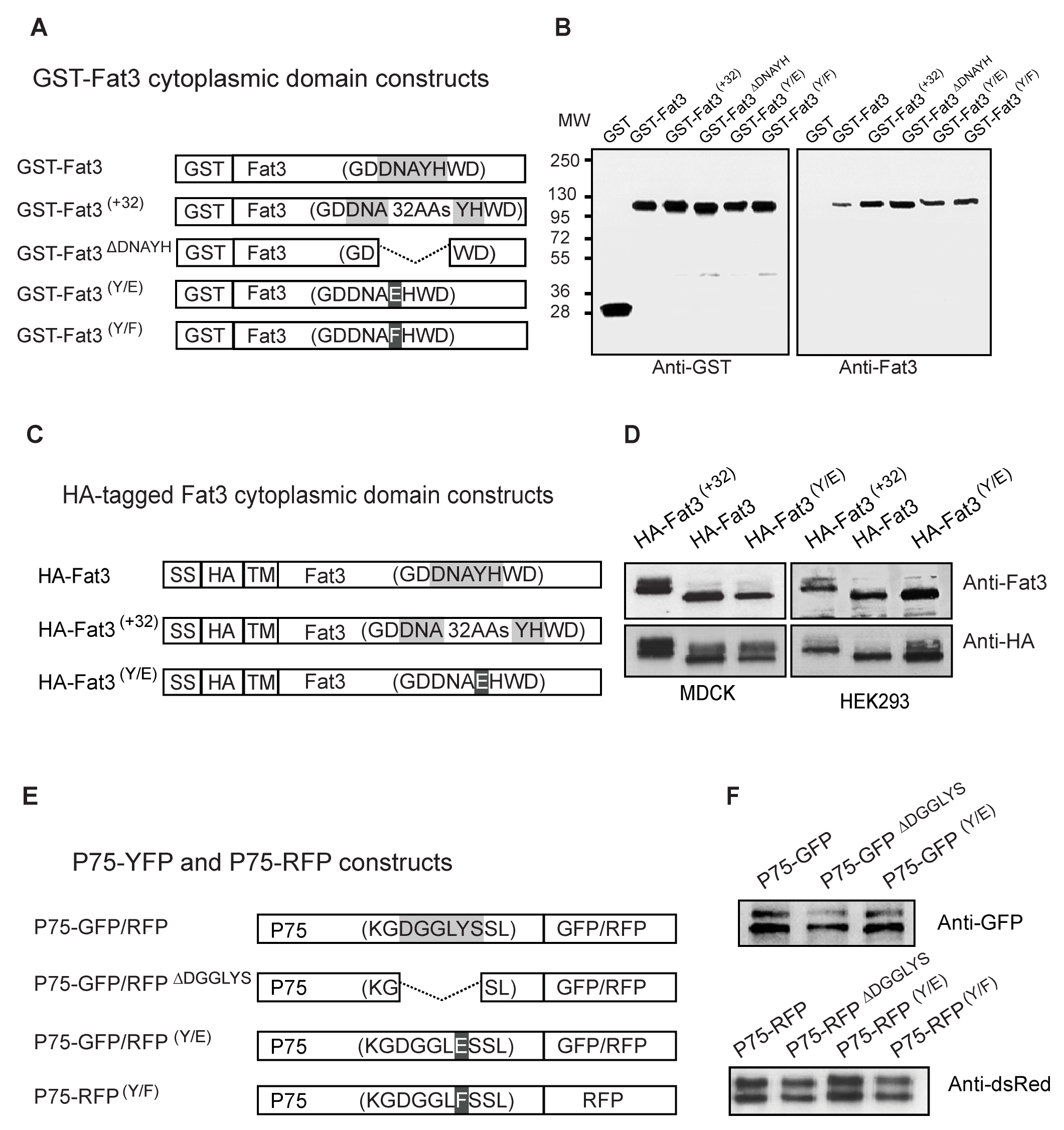

Supplement: S4 Fig — (A) Schematic representation of GST-Fat3 fusion proteins used for protein purification and binding assays in HEK293 cells. (B) Western blot of cell lysates containing GST and GST-Fat3 fusion proteins with anti-GST and anti-Fat3 antibodies. (C) Schematic representation of truncated, HA-tagged Fat3 constructs containing the Fat3 signal sequence, transmembrane and cytoplasmic domains. (D) Western Blot of cell lysates with anti-Fat3 and anti-HA antibodies showing expression of HA tagged Fat3 in MDCK and HEK293 cells. (E) Schematic representation of P75NTR constructs tagged with GFP or RFP. (F) Western Blot with anti-GFP antibody shows the expression of P75-GFP variants in MDCK cells. Western Blot using anti-dsRED antibody shows the expression of P75-RFP variants in HEK293 cells (reproduced from Fig 6A). (TIF) [file pone.0165519.s004.tif]
